# Urinary Prognostic Biomarkers in Patients With Focal Segmental Glomerulosclerosis

**DOI:** 10.5812/numonthly.16806

**Published:** 2014-03-09

**Authors:** Shiva Kalantari, Mohsen Nafar, Shiva Samavat, Mostafa Rezaei-Tavirani, Dorothea Rutishauser, Roman Zubarev

**Affiliations:** 1 Department of Basic Sciences, Faculty of Paramedical Sciences, Shahid Beheshti University of Medical Sciences, Tehran, IR Iran; 2 Department of Nephrology, Shahid Labbafinejad Medical Center, Shahid Beheshti University of Medical Sciences, Tehran, IR Iran; 3 Urology and Nephrology Research Center, Shahid Beheshti University of Medical Sciences, Tehran, IR Iran; 4 Chronic Kidney Disease Research Center, Shahid Beheshti University of Medical Sciences, Tehran, IR Iran; 5 Proteomics Research Center, Shahid Beheshti University of Medical Sciences, Tehran, IR Iran; 6 Department of Medical Biochemistry and Biophysics, Karolinska Institute, Stockholm, Sweden; 7 SciLifeLab, Stockholm, Sweden

**Keywords:** Complement Activation, Ribonuclease, Haptoglobins, Renal Dysfunction

## Abstract

**Background:**

Focal segmental glomerulosclerosis (FSGS) is a type of nephrotic syndrome which is diagnosed by renal biopsy. Degree of the proteinuria, renal dysfunction, histologic findings and the response to therapy are some factors used for evaluating the prognosis of FSGS.

**Objectives:**

In the present study, we attempted to discover some protein candidates for disease prognosis related to glomerular filtration rate (renal dysfunction).

**Patients and Methods:**

Urine samples were collected from ten patients. Urine proteome was extracted and trypsinated. Digested peptides were separated and identified by nano-flow LC-MS/MS. Protein content were determined using label-free quantification method. Protein profiles were analyzed using supervised multivariate statistical method.

**Results:**

Output of a predictive model was 54 significant proteins of which ribonuclease 2 and haptoglobin had the greatest fold change in terms of overrepresentation and underrepresentation in patients with the best and worse prognosis, respectively. Complement and coagulation cascades were the only significant pathways which were impaired in FSGS.

**Conclusions:**

Urinary biomarkers can potentially be used as non-invasive prognostic markers. However these candidate biomarkers need further validation by an alternative method and in a larger cohort.

## 1. Background

Focal segmental glomerulosclerosis (FSGS) is a type of nephrotic syndrome which is diagnosed using renal biopsy and may be found as either primary or secondary conditions. Primary FSGS occurs without an identifiable cause and the secondary type occurs in response to previous glomerular injury, glomerular hypertension, or hypertrophy (1). While the clinical presentation of FSGS is often heterogeneous, a characteristic feature of the disease is proteinuria due to loss of filtration barrier of glomeruli (2). Histological characteristics of FSGS also include scattered sclerosis of glomeruli in which only a segment of the capillary is affected (3). It is the most common cause of acquired chronic renal insufficiency in children and frequently leads to progression to the end-stage kidney disease (ESKD) (4). Factors that appear to mainly affect the prognosis include the degree of proteinuria and renal dysfunction, histologic findings, and the response to therapy. In the present study we focused on the discovery of urinary proteins responsible for developing more severe renal dysfunction as a prognostic factor using proteomics tools.

## 2. Objectives

In the present study, we attempted to discover urinary excreted proteins which can be used for the differentiation of patients with good and bad prognosis. These non-invasive biomarker candidates would be useful in the follow-up and detection of disease progression without using biopsy.

## 3. Patients and Methods

Second morning urine samples were collected from 11 patients with biopsy proven FSGS (male = 7, female = 4, mean age = 36.36), at Labbafinejad Hospital during 2011. For each patient eGFR was calculated by CKD-EPI equation at presentation. In order to study differential proteins among good and bad prognosis patients, we categorized all patients based on eGFR. Since more severe renal dysfunction at presentation is generally associated with poor renal survival (5), five patients considered as mild disease state (eGFR > 60 cc/min/1.73 m^2^) and five patients with advanced disease state and worse prognosis (eGFR < 60 cc/min/1.73 m^2^) were enrolled. Urine samples were concentrated and desalted with ultrafiltration (Millipore, Billerica, MA, USA with a 3 kDa cut off) and then were treated with acetone (up to 80% v/v), dried and re-suspended in 0.1 M ammonium acetate (pH 5). Protein concentration was then determined using the BCA (bicinchoninicacid) protein assay (Pierce, Thermo Scientific, USA) and proteins were further digested by trypsin. Digested peptides were suspended in an appropriate buffer and injected to a liquid chromatography tandem mass spectrometry (nLC-MS/MS) coupled online to a Q Exactive mass spectrometer (both-Thermo Scientific, Bremen, Germany). For details of the sample preparation protocol, MS analysis and label-free quantification procedure see the article of Kalantari et al. (6). Protein profiles then were analyzed using supervised multivariate statistical analysis. Patients were categorized based on GFR (renal dysfunction). A predictive model was then constructed and validated by 7 fold cross-validation and significant proteins were determined. Gene-set enrichment and pathway analysis on significant proteins were performed using “DAVID” software. 

## 4. Results

**Figure 1.**
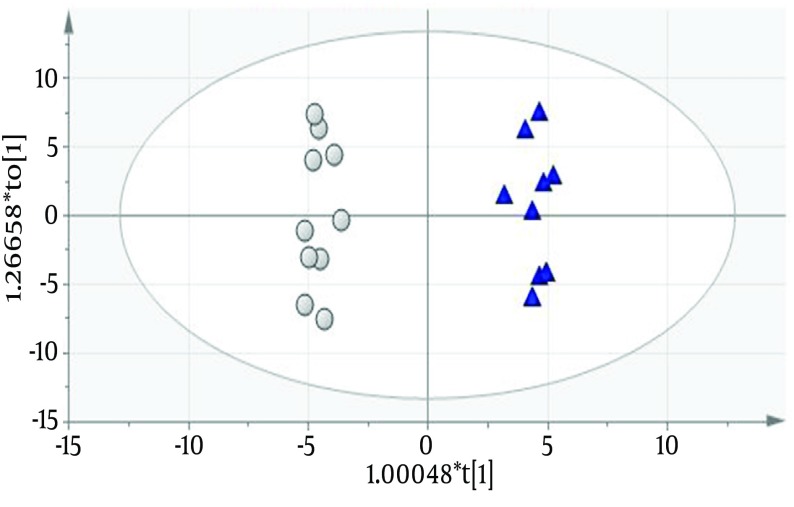
Predictive Model Constructed Using “SIMCA”. Open Circles Represent Patients With Better Prognosis and Dark Triangles Represent Patients With Worse Prognosis

Urinary protein profiles of FSGS patients using 110 protein entries were quantified as described by Kalantari et al. (7). Differential proteins between the two groups with different prognosis features (eGFR < 60 and > 60 cc/min/1.73 m^2^) were obtained using orthogonal projection to latent structures discriminant analysis (OPLS-DA) (8). A predictive model was constructed by this method (Q2 = 0.861 and R2 = 0.619) which had 100% accuracy (Figure 1). Fifty four significant biomarkers were obtained from the predictive model of which top twelve (six most positively and six most negatively correlating with GFR) are described in Table 1 as putative FSGS progression biomarkers.

**Table 1.** Top Twelve Most Significant Putative Prognostic Biomarkers for Focal Segmental Glomerulosclerosis

Protein ID	Protein Name	Biological Process	Cellular Component	Molecular Function	Fold Change (Low GFR/high GFR)	Up/Down Regulation
**RNAS2**	Ribonuclease 2	RNA catabolic process	Extracellular region/lysosome	Ribonuclease activity	7.32	↑
**CD59**	CD59 glycoprotein	Negative regulation of activation of membrane attack complex	Anchored to external side of plasma membrane/extra cellular space	Potent inhibitor of the complement membrane attack complex (MAC) action	7.21	↑
**PTGDS**	Prostaglandin-H2 D-isomerase	Prostaglandin biosynthesis/Lipid biosynthesis	Extracellular space/Golgi apparatus/rough endoplasmic reticulum	Fatty acid binding/prostaglandin-D synthase activity	5.81	↑
**B2MG**	Beta-2-microglobulin	Regulation of immune response	Extracellular space/MHC class I protein complex	Involved in the presentation of peptide antigens to the immune system	4.98	↑
**AMBP**	Alpha-1-microgolbulin	Negative regulation of immune response	Extracellular space/ plasma membrane	Serine-type endopeptidase inhibitor activity	4.39	↑
**SULF2**	Extracellular sulfatase Sulf-2	Glomerular basement membrane development	Extracellular space/plasma membrane	N-acetylglucosamine-6-sulfatase activity/calcium ion binding	4.33	↑
**CBG**	Corticosteroid-binding globulin	Glucocorticoid metabolic process/regulation of proteolysis	Extracellular space	Serine-type endopeptidase inhibitor activity/steroid binding	1.61	↓
**AFAM**	Afamin	Vitamin transport	Extracellular space	Vitamin E binding	1.64	↓
**MXRA8**	Matrix-remodeling-associated protein 8	Fibrosis process	Membrane	May play a role in the maturation and maintenance of blood-brain barrier	1.71	↓
**CO6A1**	Collagen alpha-1(VI) chain	Cell adhesion/extracellular matrix disassembly	Endoplasmic reticulum lumen/sarcolemma	Platelet-derived growth factor binding	1.74	↓
**ACTG**	Actin, cytoplasmic 2	Innate immune response/adherens junction organization	Cytoskeleton/extracellular vesicular exosome	ATP binding/structural constituent of cytoskeleton	1.76	↓
**HPT**	Haptoglobin	acute-phase response/positive regulation of cell death/response to hydrogen peroxide	extracellular space	antioxidant activity/catalytic activity	2.26	↓

## 5. Discussion

Prognosis is important to patients, clinicians, public health, and health policy makers and glomerular filtration rate (GFR) is one of the effective prognostic factors for patients with declining renal function (9, 10) and glomerular diseases. Urinary biomarkers derived from a predictive model which reflect the prognosis of glomerular diseases (based on GFR in the current study) could be considered as useful noninvasive markers for rapid, reliable and accurate diagnosis and monitoring the progression of the disease in comparison with current traditional invasive approaches, however the causes of up/down regulation of these candidates is not clear and need further experiments. Some of the most significant biomarker candidates are presented here:

RNAS2 had the greatest-fold change (7.32) as the overrepresented biomarker in patients with worse prognosis (eGFR < 60 cc/min/1.73 m^2^). A strong correlation between serum RNase levels and renal insufficiency was previously reported by Humphrey et al. (11). RNAS2 is a 3kDa protein which is found in body fluids (including urine) (12) and some tissues. The pathologic reason of its urinary elevation in glomerulosclerosis is not clear; however, to the best of our knowledge, it is reported here for the first time as a prognostic candidate marker for FSGS.

HPT (haptoglobin) is reported here as the underrepresented biomarker for FSGS progression with the greatest fold change (2.26). An association between the haptoglobin genotypes and renal function decline in individuals with long-standing type 1 diabetes was previously reported by Costacou et al. (13). The exact role of haptoglobin in progression of FSGS is not well defined and a wide targeted genomic and proteomic experimental design is required for this purpose. In the current study, some of the presented biomarkers have reported before as already known proteins implicated in glomerular disease including: B2MG (14) and AMBP (15), AFAM (6), but most of the other candidates identified in this study are novel. Gene-set enrichment analysis by “DAVID” software (16) resulted in identification of eight significant biological processes of which “acute inflammatory response” (P = 5.3 × 10^-7^), “blood coagulation” (P = 2.7 × 10^-4^) and “regulation of homeostatic process” (P = 5.3 × 10^-3^) are the more specific processes relevant to disease progression. Two of the proteins which were in the panel list (composed of 50 proteins) and were enriched in all eight significant processes were A1AG1 (orosomucoid 1) and THRB (prothrombin). We suggest these two proteins are s important proteins implicated in the pathogenesis of FSGS. Pathway analysis against KEGG database using “DAVID” software resulted in two single significant pathways which were also shown to be enriched in the previous study from our group (7) which are the complement and coagulation cascades (P = 8.2 × 10^-5^). It may suggest that impairment of complement pathway regardless of the criterion for judgment prognosis determination (either GFR or responsiveness to steroids) plays an important role in the progression and pathogenesis of FSGS. Impairment of this pathway in FSGS progression was consistent with our previous findings in which prognosis was determined based on the response to therapy.

In conclusion, a panel of urinary prognostic biomarkers was reported for FSGS. The most significant over- and underrepresented proteins in patients with worse prognosis in comparison with patients with a better prognosis were RNAS2 and HPT, respectively. These candidates were obtained from a predictive model which clustered patients based on GFR. In conclusion, involvement of proteins responsible for acute inflammatory response and also involvement of complement and coagulation pathways in disease progression were confirmed in our study using bioinformatics methods.
